# NEDD8-activating enzyme inhibition induces cell cycle arrest and anaphase catastrophe in malignant T-cells

**DOI:** 10.18632/oncotarget.28063

**Published:** 2021-09-28

**Authors:** Adam S. Kittai, Olga V. Danilova, Vi Lam, Tingting Liu, Nur Bruss, Scott Best, Guang Fan, Alexey V. Danilov

**Affiliations:** ^1^The Ohio State University, Columbus, OH 43210, USA; ^2^City of Hope Comprehensive Cancer Center, Duarte, CA 91010, USA; ^3^Oregon Health and Science University, Portland, OR 97239, USA; ^*^These authors contributed equally to this work

**Keywords:** pevonedistat, T-cell lymphoma, chromosomal instability, anaphase catastrophe

## Abstract

Peripheral T-cell lymphoma (PTCL) is characterized by poor outcomes. We and others have shown that targeting the NEDD8-activating enzyme (NAE) with an investigational inhibitor pevonedistat deregulates cell cycle and mitosis in lymphoma and leukemia. Here, we report that PTCL is characterized by increased rate of chromosomal instability. NAE inhibition promotes cell cycle arrest and induces multipolar anaphases in T-cell lymphoma cell lines, resulting in apoptosis, also observed in primary malignant PTCL cells treated with pevonedistat. We identified p27^Kip1^ as a mediator of anaphase catastrophe in these cells. Targeting neddylation with pevonedistat may be a promising approach to treatment of PTCL.

## INTRODUCTION

Peripheral T-cell lymphoma (PTCL) is an aggressive subset of non-Hodgkin lymphomas (NHL) characterized by poor outcomes [1]. Aberrant T-cell receptor and JAK-STAT signaling, genomic abnormalities targeting epigenetic modifiers and chromatin remodeling and altered cellular metabolism result in deregulated cell cycle, enhanced cell survival and proliferation in PTCL [2]. The substantial genetic and biologic heterogeneity [3] hinders success of targeted therapies in this disease and chemotherapy remains standard in both *de novo* and relapsed/refractory settings. Chromosomal instability (CIN) is predictive of poor outcomes in B-cell NHL [4] and other cancers and represents a tractable therapeutic target across multiple tumor types. We previously demonstrated that pharmacologic and genetic ablation of cyclin-dependent kinase 2 (CDK2) leads to an event termed anaphase catastrophe in lung cancer cells, confirmed with live-cell imaging [5–7]. This pathway preferentially eliminates aneuploid cancer cells by antagonizing clustering of supernumerary centrosomes during mitosis, forcing cells to undergo multipolar divisions [7]. However, it is not known if anaphase catastrophe occurs in NHL (PTCL) cells, and whether it may be induced by pharmacologic induction of endogenous CDK inhibitors in a cell.

Pevonedistat (MLN4924), an investigational small molecule inhibitor of the NEDD8-activating enzyme (NAE), demonstrated pre-clinical efficacy in B-cell NHL [8–11]. NAE ensures activation of the cullin-RING E3 ubiquitin ligases (CRLs) [12]. Pevonedistat forms a covalent adduct with NEDD8 (a ubiquitin-like modifier), resulting in NAE inhibition and accumulation of CRL substrates. CDT1, CDK inhibitors p21^Cip1^/p27^Kip1^ as well as checkpoint kinase Wee1 are among the CRL substrates which are increased following NAE inhibition. They are implicated in the anti-tumor effect of pevonedistat through induction of DNA damage, cell cycle arrest and apoptosis [8, 13, 14].

Here we investigated the effect of NAE inhibition on CIN in malignant T-cells.

## RESULTS

**Figure 1:**
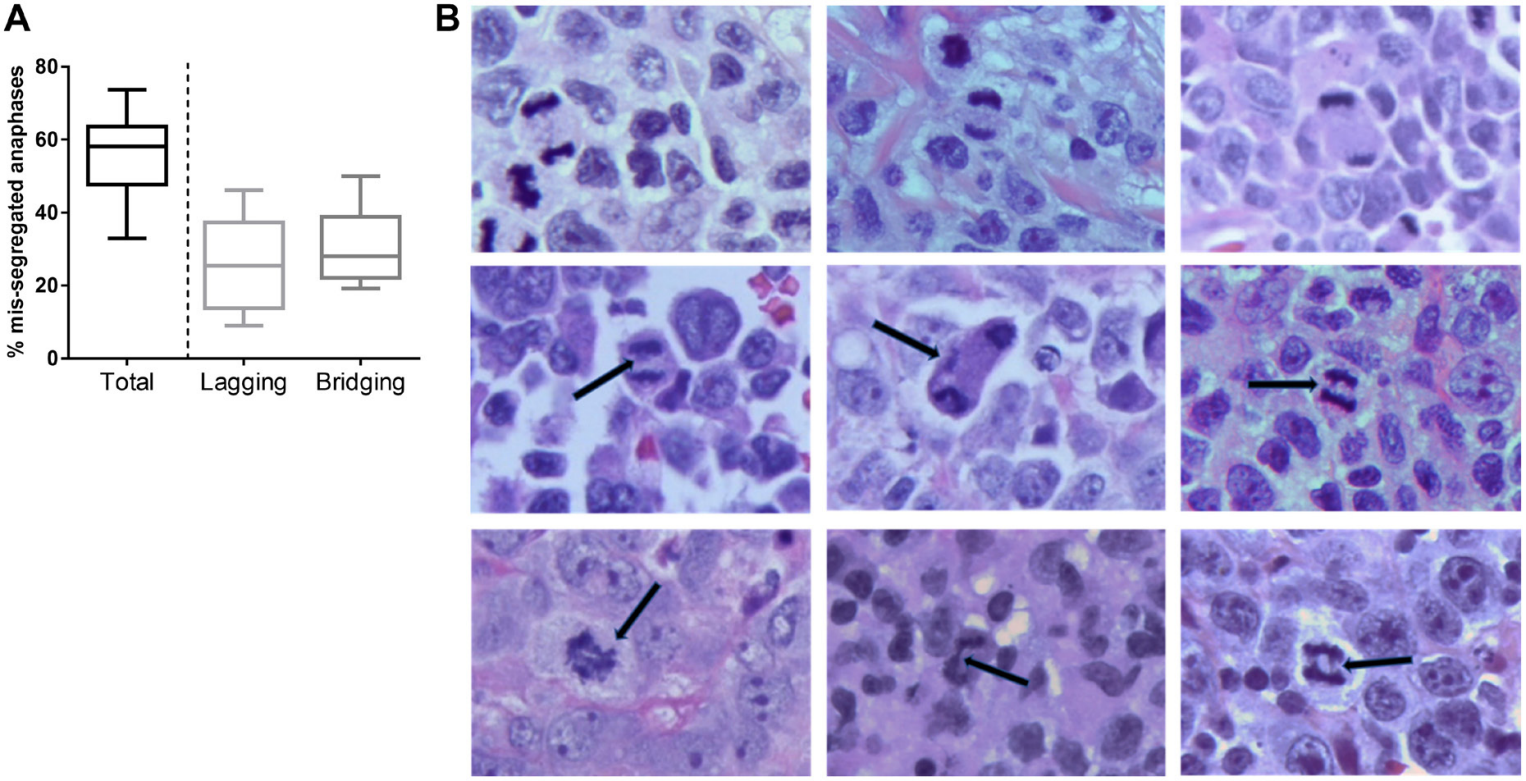
Chromosomal instability in PTCL. (**A**) A box-and-whiskers graph showing frequency of cells which exhibit mis-segregated chromosomes during anaphase (median, 25th/75th quartiles, min/max). (**B**) Images of H&E–stained T-cell lymphoma tissues showing anaphase. Representative examples of normal anaphase (top row), anaphase with lagging chromosomes (middle row), and chromatin bridges (bottom row) are shown. Scale bar, 5 μm.

First, we quantified CIN in primary lymphoid tissues from patients with PTCL. Abnormal anaphases were detected with the frequency of 56.1% ± 2.9% (Figure 1A). All tumors surveyed in this study (*N* = 14) demonstrated high level of mis-segregation using the cutoff previously established in diffuse large B-cell lymphoma (DLBCL; 31.1%) [4]. Lagging chromosomes and chromatin bridges were detected in 24.8 ± 2.9% and 31.2 ± 2.9% of anaphase cells, respectively (Figure 1A and 1B). Abnormal anaphases were not observed in reactive lymph nodes (*N* = 5). Thus, PTCL tumors exhibited profound CIN as assessed by chromosomal mis-segregation events, which exceeded that observed in DLBCL.

**Figure 2:**
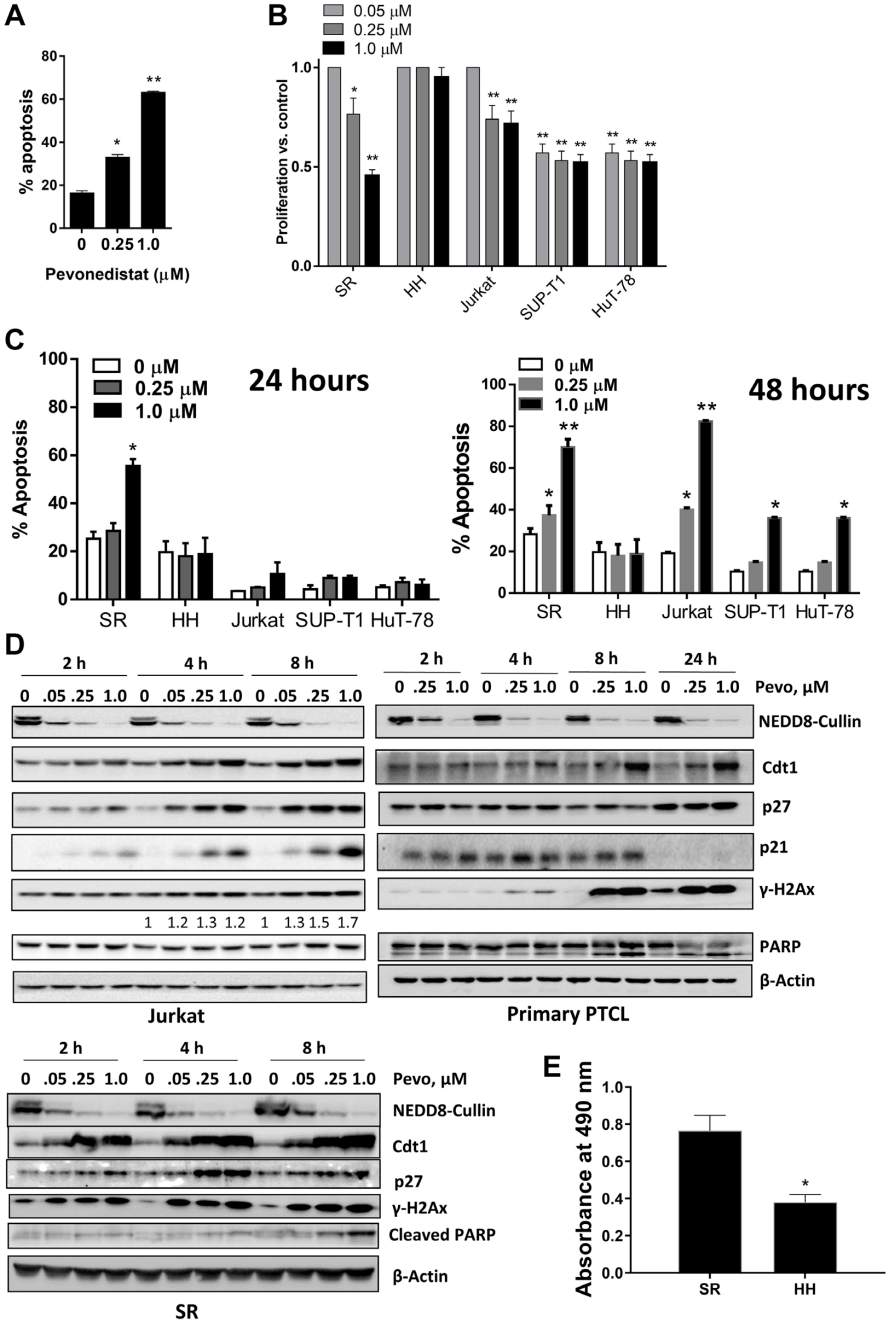
Pevonedistat induces neoplastic T cell apoptosis. (**A**) Circulating tumor cells from 3 individual patients with PTCL were treated with pevonedistat for 48 hours and assayed for apoptosis (in duplicates) in the CD3^+^ population. (**B**) Cells were incubated with the indicated concentrations of pevonedistat or vehicle control for 72 hours (6 wells per condition; performed in triplicates). Cellular proliferation was measured in a tetrazolium-based colorimetric assay. Values are normalized to vehicle-treated control. Data are the mean ± SEM. (**C**) Cells were treated with the indicated concentrations of pevonedistat for 24 (left) or 48 hours (right). Apoptosis was determined by Annexin-V staining. Data are mean ± SEM. (**D**) Jurkat, SR and primary PTCL cells were treated with pevonedistat as indicated, proteins were lysed and subjected to immunoblotting. (**E**) Cells were seeded at equal density, cultured for 72 hours and proliferation was measured in a tetrazolium-based colorimetric assay. ^*^ *p* < 0.05 and ^**^ *p* < 0.01 vs. control.

**Figure 3:**
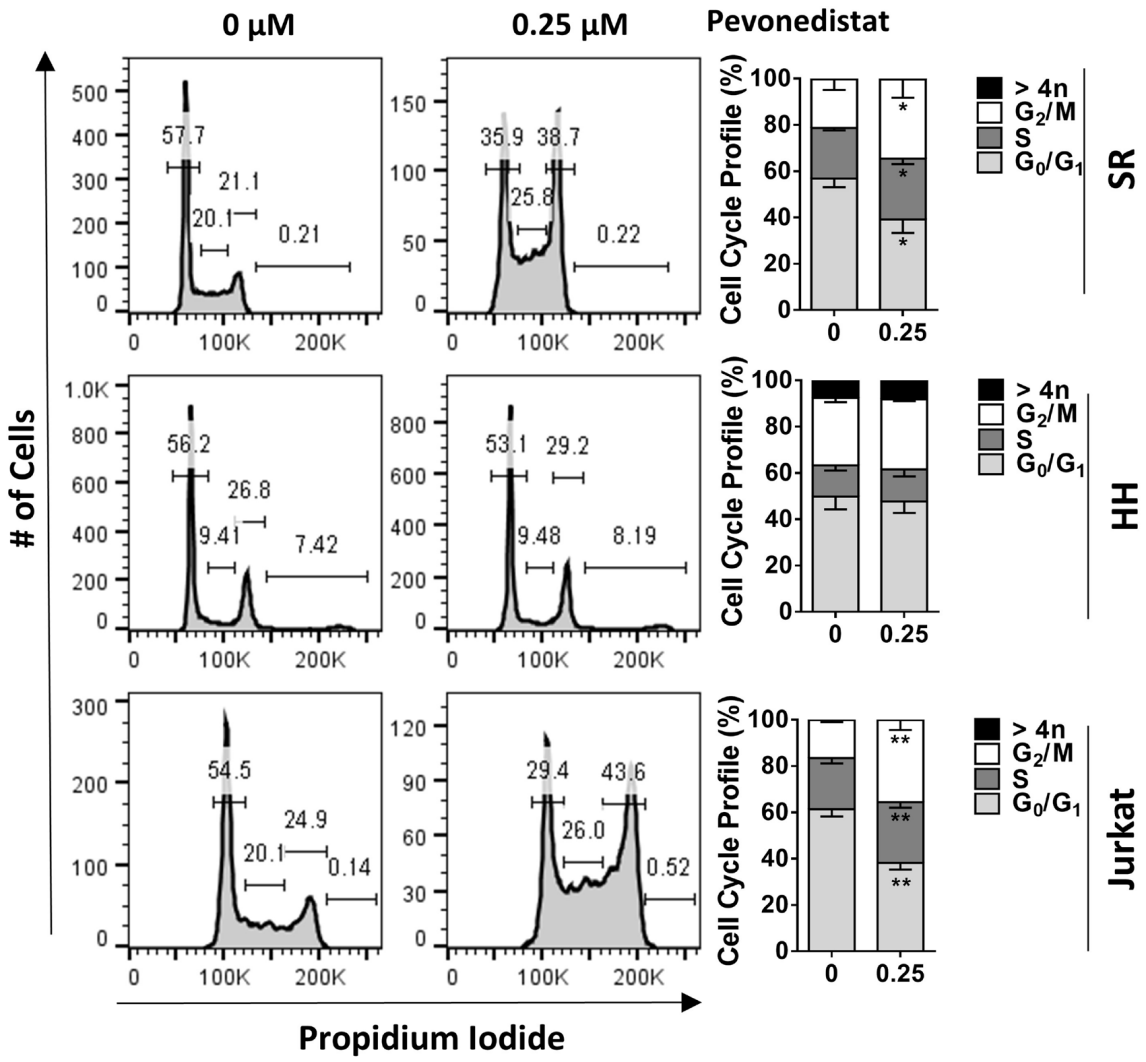
Pevonedistat induces cell cycle arrest. Cells were treated with 0.25 μM pevonedistat for 24 hours and assayed for cell cycle profiling using propidium iodide staining. ^*^ *p* < 0.05, ^**^ *p* < 0.01 vs. control.

Next, we evaluated the effect of NAE inhibition on survival and proliferation of malignant T-cells. Pevonedistat induced apoptosis of primary PTCL cells after a 24-hour exposure (Figure 2A). Pevonedistat exposure diminished proliferation of SR, Jurkat, SUP-T1 and HuT-78 cells (Figure 2B). While minimal cell line apoptosis was seen at 24 hours, 48-hour exposure to pevonedistat induced apoptosis with an IC_50_ ~0.3 μM (Figure 2C). Meanwhile, HH cells were resistant. Treatment with pevonedistat disrupted cullin neddylation in a dose-dependent manner (Figure 2D). We observed rapid increases of CRL substrate proteins, including CDT1, p21^Cip1^ and p27^Kip1^ (hereinafter p27) following NAE inhibition. HH cells and primary PTCL cells showed weak induction of CDT1, consistent with their low proliferation rate (Figure 2E). Pevonedistat induced DNA damage as evidenced by an increase in γH2AX (Figure 2D). SR and Jurkat cells, but not HH cells, exhibited arrest in S and G_2_/M phases of cell cycle when treated with pevonedistat (Figure 3).

**Figure 4:**
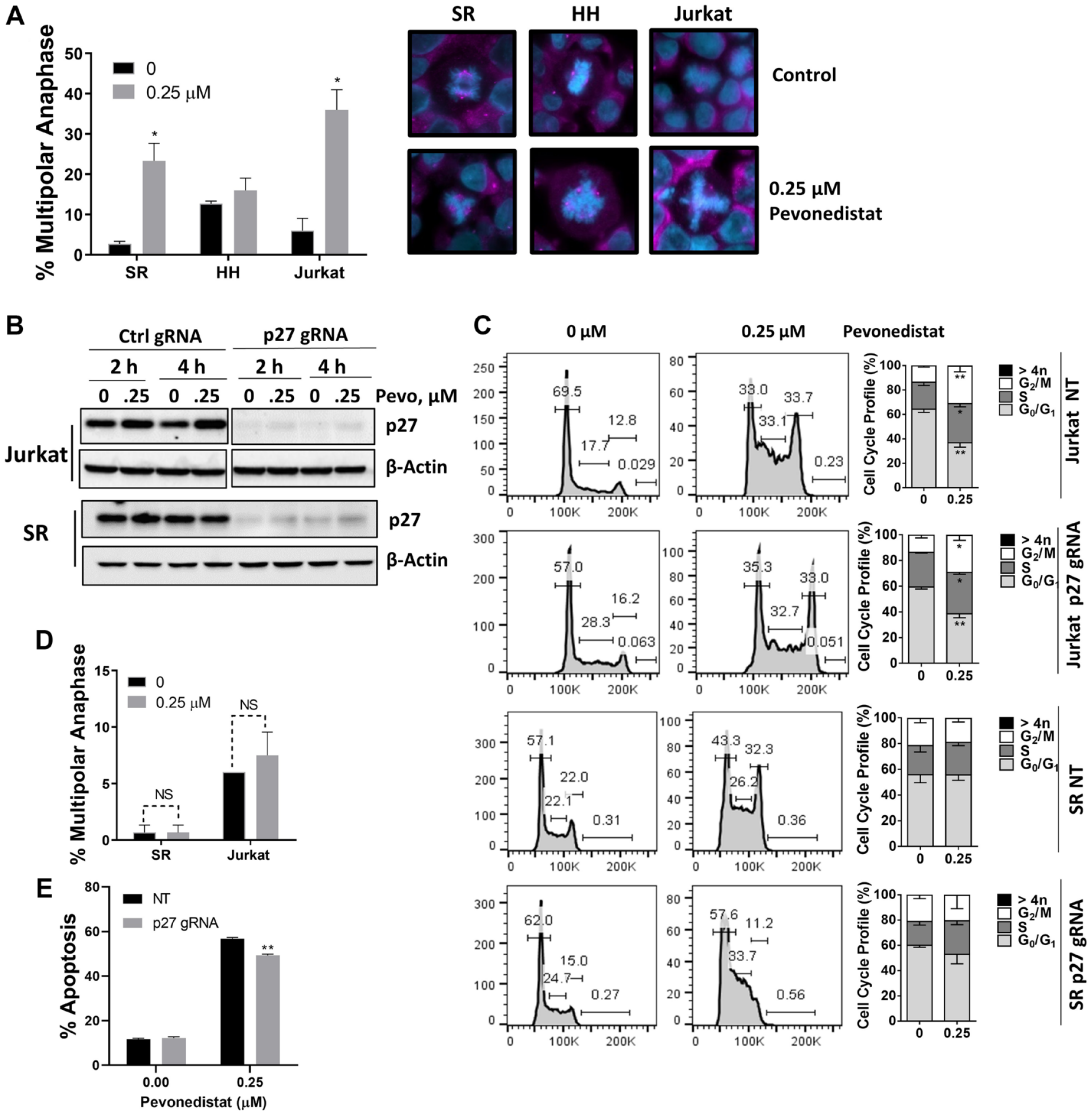
Malignant T-cells undergo anaphase catastrophe following NAE inhibition. (**A**) Cells were incubated with the indicated concentration of pevonedistat or vehicle control for 24 hours. Cells were immunostained with γ-tubulin (pink) and counterstained with DAPI (blue). Total anaphases were counted (50) and the proportion of multipolar anaphases is shown. Data are the mean ± SEM of at least 3 independent experiments. Representative examples of normal mitosis and multipolar anaphase are shown. (**B**) Jurkat and SR cells were transfected with a lentiviral CRISPR toolbox to achieve knockout of p27, or vector control. Cells were treated with pevonedistat as indicated, proteins were lysed and subjected to immunoblotting. (**C**) Cells were treated with pevonedistat for 24 hours and subjected to cell cycle profiling with propidium iodide staining. A summary of three independent experiments is shown. (**D**) p27-deficient cells were incubated with 0.25 μM pevonedistat or vehicle control for 24 hours. Cells were immunostained with γ-tubulin and counterstained with DAPI. Total anaphases were counted (50) and the proportion of multipolar anaphases is shown. Data are the mean ± SEM of six (Jurkat) and three (SR) independent experiments. (**E**) Jurkat cells were treated with 0.25 μM pevonedistat or vehicle control for 48 hours. Apoptosis was determined by Annexin-V staining. Data are mean ± SEM of 6 independent experiments. ^*^ *p* < 0.05 and ^**^ *p* < 0.01 vs. control.

Since p27 (an endogenous CDK inhibitor) accumulated in malignant T-cells treated with pevonedistat, we studied anaphase catastrophe in this setting. We observed a significant induction of anaphase catastrophe in SR and Jurkat cells, but not HH cells, following NAE inhibition (Figure 4A). Upon 24-hour treatment with 0.25 μM pevonedistat, 36.0 ± 5.0% of Jurkat and 23.3 ± 4.4% of SR cells exhibited multipolar anaphases, compared with 6.0 ± 3.1% and 2.0 ± 0.7% with vehicle control, respectively. Thus, induction of anaphase catastrophe preceded apoptosis in those cells.

To evaluate whether induction of p27 is involved in mitotic deregulation in this context, we employed CRISPR-Cas9 editing. Introduction of *CDKN1B* gRNA resulted in complete loss of p27 protein in both Jurkat and SR cell lines (Figure 4B). p27-deficient cells continued to exhibit arrest in S/G_2_/M phases of cell cycle (Figure 4C), suggesting that this effect is likely Cdt1-mediated as previously shown in other model systems [8, 15]. However, SR and Jurkat cells lacking p27 no longer exhibited anaphase catastrophe following treatment with pevonedistat (Figure 4D). Furthermore, p27-deficient cells demonstrated slightly diminished susceptibility to pevonedistat-induced apoptosis (Figure 4E). This indicates that p27 accumulation following NAE inhibition contributes to anaphase catastrophe and may in part mediate apoptosis in malignant T-cells.

## DISCUSSION

While we previously reported that lung cancer cells undergo anaphase catastrophe when exposed to chemotherapy and CDK2 inhibitors [5, 6], here we show that this pathway is also induced in neoplastic T-cells. p27 complexes with CDK2-Cyclin E/A, thus restraining progression through G_1_/S phase of the cell cycle [16]. We now also demonstrate an additional role, where following NAE inhibition p27 mediates anaphase catastrophe. We did not explore whether this effect is mediated via attenuated activity of CDK2. It has been recognized that in addition to CDK2-Cyclin E/A, p27 has complex interplay with other CDK complexes, including CDK1 and CDK4/6 [16, 17]. While loss of CDK1 is also known to mediate anaphase catastrophe, potential role of CDK4/6 in this setting has not been explored [6]. Furthermore, we have not investigated other CRL substrates which could potentially be involved in this mechanism, such as p21^Cip1^, which can attenuate activity of multiple CDK complexes, or proteins involved in regulation of DNA damage checkpoint, i.e. CDT1 and Wee1. Future studies should also determine if pevonedistat would synergize with chemotherapy to induce anaphase catastrophe.

In sum, we demonstrate that PTCL exhibits pronounced chromosomal mis-segregation. Targeting NAE with pevonedistat leads to cell cycle arrest and anaphase catastrophe in neoplastic T-cells. This effect, possibly coupled with immunomodulatory activity of pevonedistat recently described by our group [18], justifies continued exploration of pevonedistat as a novel therapeutic approach in T-cell NHL.

## MATERIALS AND METHODS

### Cells, cell cycle and apoptosis

Following approval by the IRB, PTCL cells were isolated from PBMC of three patients with high circulating tumor burden using standard Ficoll–Hypaque technique (>95% CD3^+^/CD5^–^ tumor cells). SR, Jurkat and SUP-T1 (T-lymphoblast) cell lines and Sezary HH and HuT-78 cells were obtained from the American Type Culture Collection (ATCC). All cells were cultured in RPMI-1640 medium supplemented with 15% fetal bovine serum.

To measure cell proliferation, cells were plated in 96-well plates at 3000/well in 100 μL (6 per sample) and incubated for 72 hours. Viable cells were measured using a CellTiter Aqueous One Solution Cell Proliferation Assay (Promega).

Cell apoptosis was measured in duplicates using the ApoScreen Annexin V Apoptosis Kit as previously described (Southern Biotech) [8]. For cell cycle analysis, 2 × 10^5^ cells were fixed in ice cold 70% ethanol while being vortexed, incubated on ice for 15 minutes, washed in PBS and resuspended in 250 μl of staining solution containing 20 ng/ml propidium iodide, 200 ng/ml RNAse A (Sigma Aldrich), 0.1% Triton-X 100 and 1 μl CD19-FITC mAb in PBS. Cells were incubated for 15 minutes and submitted to flow cytometry. Analysis was performed using FlowJo software (Tree Star).

Pevonedistat was provided by Millennium Pharmaceuticals, Inc. (Cambridge, MA, USA), a wholly owned subsidiary of Takeda Pharmaceutical Company Limited.

### Immunoblotting

Cells were lysed in RIPA buffer with supplements and proteins were analyzed by immunoblotting as previously described [8]. The following antibodies were used: NEDD8, p21^Cip1^, p27^Kip1^, CDT1, γ-H2AX, PARP and cleaved PARP, β-Actin, and horseradish peroxidase-conjugated anti-mouse and anti-rabbit antibodies (Cell Signaling Technologies). In each case, a representative image of at least 3 independent immunoblotting experiments is shown.

### CRISPR-Cas9 genome editing

A lentiCRISPRv2 system (Addgene, 62988) was used as described in GeCKO lentiviral CRISPR toolbox [19]. Lentivirus was packaged using psPAX2 (Addgene #12260) and VSVG (Thermo Fisher, K497500) plasmids in HEK293T cells (ATCC) using jetPRIME transfection reagent (Polyplus Transfection). Viral supernatants were collected at 48 and 60 hours and quantified with qPCR Lentivirus Titration Kit (Applied Biological Materials Inc). Lentivirus was transduced in 1 × 10^6^ Jurkat and HH cells at a MOI of 10 using spinoculation with 2500 RPM for 90 min at 30°C. Infected cells were selected in 2 μg/mL puromycin (Gibco) 2 days post transduction. *CDKN1B* (p27^Kip1^) gRNA oligos: forward 5′-CACCGCAGGAACCTCTTCGGCCCGG(TGG)-3′, reverse 5′-AAACCCGGGCCGAAGAGGTTCCTGC-3′.

### Anaphase catastrophe

Cells were fixed in 10% formalin, stained with anti-γ-tubulin–specific antibody (Thermo Fisher), and independently mounted with Pro-Long Gold antifade reagent supplemented with 40,6-diamidino-2-phenylindole (DAPI; Thermo Fisher). Fluorescent images were captured with an F-view II monochrome camera (Olympus, U-CMAD3) mounted on Zeiss Apotome 2. Total anaphase cells were counted and those that contained ≥ 3 spindle poles were scored as multipolar.

### Chromosomal mis-segregation in lymphoid tissues

Fourteen patients with PTCL (6 anaplastic large cell lymphoma, 3 angioimmunoblastic T-cell lymphoma, one NK/T-cell lymphoma and 4 PTCL NOS) were included in the study per the local IRB regulations. Formalin-fixed paraffin-embedded samples were stained with H&E. Cells undergoing anaphase were surveyed for evidence of chromosome mis-segregation as previously reported [4]. An average of 59 (range, 20 to 100) anaphases were scored per sample. Normal anaphase was defined by the absence of any chromatin staining between the chromosome masses, while mis-segregation was defined by the existence of either lagging chromosomes (an area of hematoxylin staining completely isolated in between the remaining segregating chromosomes during anaphase) or chromatin bridges (at least one continuous band of hematoxylin staining linking the segregating chromosomes).

### Statistical analysis

Statistical analysis was performed with Student *t* test in GraphPad Prism software. ^*^ *p* < 0.05 and ^**^ *p* < 0.01 throughout the manuscript. All experiments were performed at least in biological triplicates.
